# Complement System Inhibition Modulates the Pro-Inflammatory Effects of a Snake Venom Metalloproteinase

**DOI:** 10.3389/fimmu.2019.01137

**Published:** 2019-05-22

**Authors:** Lygia Samartin Gonçalves Luchini, Giselle Pidde, Carla Cristina Squaiella-Baptistão, Denise V. Tambourgi

**Affiliations:** Immunochemistry Laboratory, Butantan Institute, São Paulo, Brazil

**Keywords:** *Bothrops pirajai*, SVMP, complement system, whole blood model, compstatin

## Abstract

Envenomation by *Bothrops* snakes causes prominent local effects, including pain, oedema, local bleeding, blistering and necrosis, and systemic manifestations, such as hemorrhage, hypotension, shock and acute renal failure. These snake venoms are able to activate the complement system and induce the generation of anaphylatoxins, whose mechanisms include the direct cleavage of complement components by snake venom metalloproteinases and serine proteinases present in the venoms. A metalloproteinase able to activate the three complement pathways and generate active anaphylatoxins, named C-SVMP, was purified from the venom of *Bothrops pirajai*. Considering the inflammatory nature of *Bothrops* venoms and the complement-activation property of C-SVMP, in the present work, we investigated the inflammatory effects of C-SVMP in a human whole blood model. The role of the complement system in the inflammatory process and its modulation by the use of compstatin were also investigated. C-SVMP was able to activate the complement system in the whole blood model, generating C3a/C3a desArg, C5a/C5a desArg and SC5b-9. This protein was able to promote an increase in the expression of CD11b, CD14, C3aR, C5aR1, TLR2, and TLR4 markers in leukocytes. Inhibition of component C3 by compstatin significantly reduced the production of anaphylatoxins and the Terminal Complement Complex (TCC) in blood plasma treated with the toxin, as well as the expression of CD11b, C3aR, and C5aR on leukocytes. C-SVMP was able to induce increased production of the cytokines IL-1β and IL-6 and the chemokines CXCL8/IL-8, CCL2/MCP-1, and CXCL9/MIG in the human whole blood model. The addition of compstatin to the reactions caused a significant reduction in the production of IL-1β, CXCL8/IL-8, and CCL2/MCP-1 in cells treated with C-SVMP. We therefore conclude that C-SVMP is able to activate the complement system, which leads to an increase in the inflammatory process. The data obtained with the use of compstatin indicate that complement inhibition may significantly control the inflammatory process initiated by *Bothrops* snake venom toxins.

## Introduction

Snakebite envenoming was recently included in the World Health Organization (WHO) list of neglected tropical diseases due to its high incidence and severity (1). Approximately 1.8 to 2.7 million cases of human envenoming and 94,000 to 125,000 deaths are estimated every year worldwide (2, 3).

Snake venoms are complex mixtures of different compounds, including proteins with or without enzymatic activity, whose proportion varies between different genera, resulting in distinct clinical manifestations. The complement system is one of the various targets that can be affected by the action of snake venoms from the Elapidae and Viperidae families. Enzymatic cleavage of C3 and C5 components directly by venom toxins or indirectly by complement activation results in C3a and C5a release, which exerts strong pro-inflammatory effects, contributing to the envenoming symptoms (4).

Belonging to the Viperidae family, the *Bothrops* genus comprises more than 20 species in the Americas, the majority of which are classified as Category 1 by the WHO, which means they are “highly venomous snakes that are common or widespread and cause numerous snakebites, resulting in high levels of morbidity, disability or mortality” (5). Envenomation by *Bothrops* snakes causes prominent local effects, including pain, oedema, local bleeding, blistering and necrosis, and systemic manifestations, such as hemorrhage, hypotension, shock, and acute renal failure. The local and systemic effects of *Bothrops* envenomation are closely related to the presence of certain classes of toxins in the venoms, such as snake venom metalloproteinases (SVMPs), serine proteinases, phospholipases A_2_ (PLA_2_s), C-type lectins, L-amino acid oxidases (LAO), and hyaluronidases (6, 7), among which there are toxins able to activate the complement system (4), contributing to the inflammatory symptoms observed in envenomed patients.

Analyzing the venoms of 19 species of *Bothrops* snakes present in the Brazilian territory, our group described their PLA_2_, hyaluronidase and proteolytic activities (8), as well as their ability to activate the human complement system *in vitro* (9). These venoms were able to activate classical, alternative and/or lectin complement pathways and induce the generation of anaphylatoxins, whose mechanisms included the direct cleavage of complement components by SVMPs and serine proteinases present in the venoms (9). Using the ability to cleave the complement component C3 as a screening reaction, our group subsequently purified and characterized a P-I class SVMP from the venom of *Bothrops pirajai*, named C-SVMP, that is able to activate the three complement pathways by cleaving the complement components C3, C4, and C5, generating active anaphylatoxins (6). This *B. pirajai* venom P-I class SVMP was also purified by another group (10), and it was shown to exert local inflammatory effects *in vivo* (11), which might be related to its anticomplementary activity. Other toxins from the venom of *B. pirajai* have also been implicated in the activation of the complement system, as demonstrated for two serine proteinases (12) and one LAO (13), although their effects on the complement system have been considered weak by the authors.

In 2002, Mollnes et al. described an *in vitro* human whole blood model to study the role of the complement system in sepsis (14). The anticoagulant used in this model is lepirudin, a recombinant form of hirudin derived from leeches that acts on thrombin and does not affect complement system activity (15). This model allows for the analysis of the role of complement activation in the production of inflammatory cytokines and chemokines and in the expression of activation molecules by leukocytes (14, 16). In addition, this model can be used to interfere with the complement system through the use of specific inhibitors, such as compstatin. Compstatin is a cyclic peptide that prevents the proteolytic cleavage of C3 by C3 convertase, inhibiting the activation of classical and alternative complement pathways (17). This molecule has potential clinical applications and has been used in several models (18, 19), including the human whole blood model (14).

Our group recently used the human whole blood model and the complement inhibitor compstatin to study the endotoxic-like shock induced by sphingomyelinase D, the main toxin present in the venom of *Loxosceles* spiders (20). Therefore, it is a useful human model to determine the role of the complement system in the systemic inflammatory events triggered by animal venoms or isolated toxins.

Considering the inflammatory nature of *Bothrops* venoms and the complement-activation property of the C-SVMP from *B. pirajai*, in the present work, we investigated the inflammatory effects of C-SVMP in the human whole blood model. The role of the complement system in the inflammatory process and its modulation by the use of compstatin were investigated, aiming to open new perspectives for the use of complement inhibitors as complementary therapy in the treatment of accidents by venomous animals.

## Materials and Methods

### *B. pirajai* Venom and C-SVMP Purification

The access to the venom of *B. pirajai* was submitted to the National System of Management of Genetic Heritage and Associated Traditional Knowledge (SisGen) under the register number AD4FE4C.

Lyophilized *B. pirajai* venom was provided by the Herpetology Laboratory of the Butantan Institute (São Paulo, SP, Brazil) and stored at −20°C. Venom was reconstituted in sterile non-pyrogenic saline at 1 mg/mL, and aliquots were maintained at −80°C until use. The protein concentration of reconstituted venom was determined using the Pierce® BCA Protein Assay Kit (Thermo Fisher Scientific, IL, USA) according to the manufacturer's instructions.

C-SVMP from *B. pirajai* venom was purified by two-step molecular exclusion chromatography using the ability to cleave the purified human C3 complement component as the criterion for fraction selection, as previously described (6). The final protein concentration was determined using the Pierce® BCA Protein Assay Kit (Thermo Fisher Scientific), according to the manufacturer's instructions. The identity of purified toxin was confirmed by mass spectrometry, and its proteolytic activity was confirmed by fluorometric assay, as previously described (6). The presence of endotoxin contamination in purified C-SVMP was determined using a PYROGENT^TM^ Plus Gel Clot LAL Assay (Lonza, MD, USA), kindly performed by the Microbiological Control Section of the Butantan Institute. The concentration of endotoxin in purified C-SVMP was below the test sensitivity (< 0.125 EU/mL).

### Human Whole Blood Model

This study was approved by the Human Research Ethics Committee from the Biomedical Sciences Institute of the University of São Paulo (São Paulo, SP, Brazil) under the certificate number 1145/CEP.

The human whole blood model described by Mollnes et al. (14) was used. Blood samples from healthy consenting donors were collected by venepuncture into tubes containing 50 μg/mL of lepirudin (Refludan®, Celgene, NJ, USA), the recombinant form of hirudin, a thrombin-inhibitor anticoagulant that does not interfere with the complement cascade. Immediately after collection, 720 μL of the blood samples were incubated with 280 μL of PBS (8.1 mM Na_2_HPO_4_, 1.5 mM KH_2_PO_4_, 137 mM NaCl, 2.7 mM KCl, pH 7.4), LPS (lipopolysaccharide from *Escherichia coli* strain O111:B4, Sigma-Aldrich, MI, USA; 5 μM final concentration) or C-SVMP (1 μM final concentration) for 30 minutes in a water bath at 37°C under agitation. Under these conditions, C-SVMP did not promote blood coagulation (data not shown), allowing for subsequent analyses of plasma and leukocytes, as described below.

### Blood Treatment With Compstatin or Control Peptide

Compstatin is a 13-amino acid cyclic peptide (ICVVQDWGHHRCT) isolated from a phage-displayed random peptide library that binds to C3, inhibiting its proteolytic cleavage by C3 convertase. The 50% inhibitory concentrations (IC_50_s) required for inhibition of classical and alternative pathway-mediated haemolytic activities for compstatin are 63 and 12 μM, respectively. Reduction and alkylation of compstatin results in a control peptide (IAVVQDWGHHRAT), which completely lacks complement-inhibitory activity (17).

Herein, 720 μL of the blood samples were pre-incubated with 140 μL of diluent buffer (PBS), compstatin (Tocris Bioscience, Bristol, UK; 200 μM final concentration) or control peptide (Tocris Bioscience; 200 μM final concentration) for 30 min at 37°C. Then, 140 μL of PBS, LPS (5 μM final concentration) or C-SVMP (1 μM final concentration) were added and incubated for 30 min in a water bath at 37°C under agitation. Plasma and leukocytes were processed for analysis, as described below.

### Detection of Anaphylatoxins, Soluble C5b-9 Complex, Cytokines, and Chemokines in Plasma Samples

Whole blood samples were centrifuged at 405 x *g* for 10 min at 4°C. Plasma samples were collected, and EDTA (ethylenediaminetetraacetic acid, Sigma-Aldrich; 10 mM final concentration) was added to stop further complement activation. Aliquots were stored at −80°C until analysis.

Plasma levels of C3a/C3a desArg and C5a/C5a desArg were determined using the OptEIA^TM^ Human C3a and C5a ELISA Kits (BD Biosciences, CA, USA). Plasma levels of the soluble C5b-9 complex (TCC) were determined using the MicroVue^TM^ SC5b-9 Plus EIA Kit (Quidel Corporation, CA, USA). The production of IL-12p70, TNF-α, IL-10, IL-6, and IL-1β was determined using the BD^TM^ Cytometric Bead Array (CBA) Human Inflammatory Cytokines Kit (BD Biosciences). The production of CXCL10/IP-10, CCL2/MCP-1, CXCL9/MIG, CCL5/RANTES and CXCL8/IL-8 was determined using the BD^TM^ CBA Human Chemokine Kit (BD Biosciences). All assays were performed according to the manufacturers' instructions.

### Analysis of Surface Molecule Expression by Leukocytes

Whole blood samples were incubated with BD FACS^TM^ Lysing Solution (BD Biosciences) according to the manufacturer's instructions, centrifuged at 405 x *g* for 10 min at 4°C, and resuspended in FACS Fixing Solution (PBS containing 0.5% paraformaldehyde). Cells were then stained with the following specific mouse anti-human monoclonal antibodies or isotype controls labeled with fluorescein isothiocyanate (FITC), R-phycoerythrin (PE), allophycocyanin (APC), PE-cyanine 7 (PE-Cy7), APC-Cy7, or Alexa Fluor 647 (A647): anti-CD11b PE (clone VIM12, Invitrogen, Thermo Fisher Scientific, MA, USA), anti-CD14 FITC (clone TüK4, Invitrogen, Thermo Fisher Scientific), anti-CD14 FITC (clone 61D3, eBioscience, CA, USA), anti-TLR2 PE (clone TL2.1, eBioscience), anti-TLR4 PE (clone HTA125, eBioscience), anti-C3aR PE (clone 17, Santa Cruz Biotechnology, TX, USA), anti-C5aR1 FITC (clone 8D6, Santa Cruz Biotechnology), anti-CD33 APC (clone WM53, BD Biosciences), anti-CD66b A647 (clone G10F5, BD Biosciences), mouse IgG1κ PE isotype control (Dako, CPH, Denmark) and mouse IgG2aκ FITC isotype control (Dako). After the incubation of samples for 30 min at room temperature in the dark, the acquisition volume was completed with FACS buffer (PBS containing 1% BSA and 0.01% sodium azide), and samples were maintained at 4°C for up to 30 min before the acquisition.

Fluorescence data were acquired using a BD FACSCanto II flow cytometer (BD Biosciences), and results were analyzed using BD FACS Diva Software v. 4.1 (BD Biosciences). Monocytes and granulocytes were gated based on their forward- and side-scatter features, followed by selection of CD33+ and CD66b+ populations, respectively. Expression of CD11b, CD14, TLR2, TLR4, C3aR, and C5aR1 was determined by the median fluorescence intensity (MFI) of stained cells after subtraction of the respective isotype control.

### Statistical Analysis

Data were analyzed by two-way ANOVA followed by Bonferroni's post-test using GraphPad Prism software v.5.1 (GraphPad Software, CA, USA). The differences were considered significant at *p* < 0.05.

## Results

### C-SVMP Activates the Complement System in the Human Whole Blood Model

**Figure 1:**
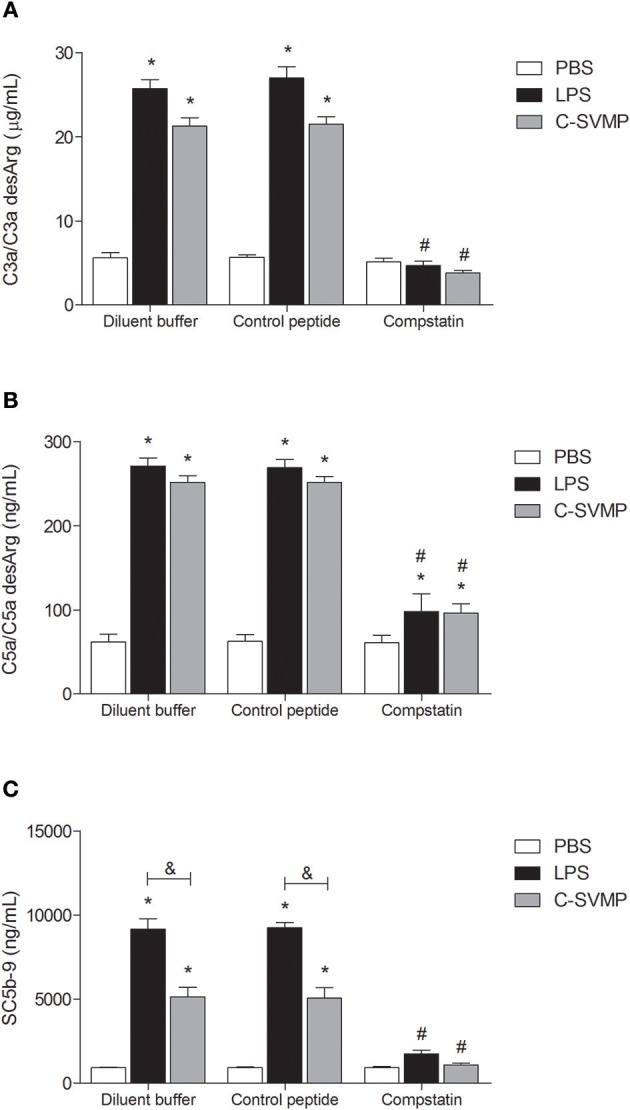
Complement activation by C-SVMP in the human whole blood model. Blood samples were collected in the presence of lepirudin and pre-incubated with diluent buffer, compstatin or control peptide for 30 min. Then, PBS, LPS or C-SVMP was added, and the samples were further incubated for 30 min. Plasma was collected and processed, and EDTA was added to stop further complement activation. Plasma levels of C3a/C3a desArg **(A)**, C5a/C5a desArg **(B)** and soluble C5b-9 complex **(C)** were determined using the OptEIA^TM^ Human C3a and C5a ELISA Kits and MicroVue^TM^ SC5b-9 Plus EIA Kit, respectively, according to the manufacturers' instructions. Data represent the mean ± S. D. of three independent experiments. *p < 0.05 compared to the respective PBS treatment. &*p* < 0.05 between the LPS and C-SVMP treatments. #*p* < 0.05 between the compstatin and diluent buffer pretreatments.

Incubation of human blood with LPS (positive control) or C-SVMP resulted in a significant increase in C3a/C3a desArg (Figure 1A), C5a/C5a desArg (Figure 1B) and SC5b-9 (Figure 1C) plasma levels compared to those from samples incubated with PBS, indicating activation of the complement system. C-SVMP induced similar levels to those induced by LPS of C3a/C3a desArg (Figure 1A) and C5a/C5a desArg (Figure 1B) but lower levels of SC5b-9 (Figure 1C).

Pre-incubation of whole blood with compstatin resulted in total inhibition of C3a/C3a desArg (Figure 1A) and SC5b-9 (Figure 1C) generation and partial inhibition of C5a/C5a desArg generation (Figure 1B) compared to that observed after pre-incubation with diluent buffer. The effect of compstatin was confirmed to be specific since pre-incubation with its control peptide did not affect complement system activation (Figure 1).

### C-SVMP Activates Blood Monocytes and Granulocytes in a Partially Complement-Dependent Manner

**Figure 2:**
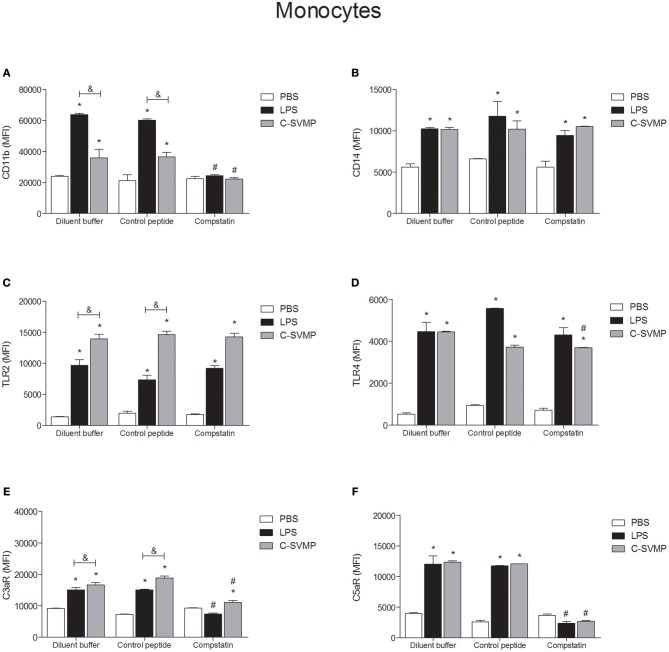
Partially complement-dependent activation of monocytes by C-SVMP in the human whole blood model. Blood samples were collected in the presence of lepirudin and pre-incubated with diluent buffer, compstatin or control peptide for 30 min. Then, PBS, LPS or C-SVMP was added, and the samples were further incubated for 30 min. After erythrocyte lysis, leukocytes were fixed, stained with specific mouse anti-human monoclonal antibodies or isotype controls that were fluorochrome labeled and incubated for 30 min. Samples were analyzed by flow cytometry. Monocytes were gated based on their forward- and side-scatter features, followed by selection of the CD33^+^ population. The expression of CD11b **(A)**, CD14 **(B)**, TLR2 **(C)**, TLR4 **(D)**, C3aR **(E)**, and C5aR1 **(F)** was determined by the median fluorescence intensity (MFI) of stained cells after subtraction of the respective isotype control. Data represent the mean ± S. D. of four independent experiments. *p < 0.05 compared to the respective PBS treatment. &*p* < 0.05 between the LPS and C-SVMP treatments. #*p* < 0.05 between the compstatin and diluent buffer pretreatments.

C-SVMP induced the activation of blood monocytes, as demonstrated by the upregulation of CD11b, CD14, TLR2, TLR4, C3aR, and C5aR1 expression (Figure 2). Interestingly, the effects of C-SVMP on these cells were slightly different from the effects of LPS since LPS induced higher expression than C-SVMP of CD11b (Figure 2A) but lower expression of TLR2 (Figure 2C) and C3aR (Figure 2E). Pre-incubation with compstatin completely inhibited the upregulation of CD11b (Figure 2A) and C5aR1 (Figure 2F) by C-SVMP and LPS but exerted no effect on the expression of CD14 (Figure 2B) and TLR2 (Figure 2C). Again, slight differences were observed when comparing the effects of compstatin on the C-SVMP and LPS treatments. Compstatin partially inhibited the induction of expression of TLR4 by C-SVMP but exerted no effect on its induction by LPS treatment (Figure 2D). However, compstatin completely inhibited the upregulation of C3aR by LPS treatment but only exerted a partial effect on its induction by C-SVMP treatment (Figure 2E).

**Figure 3:**
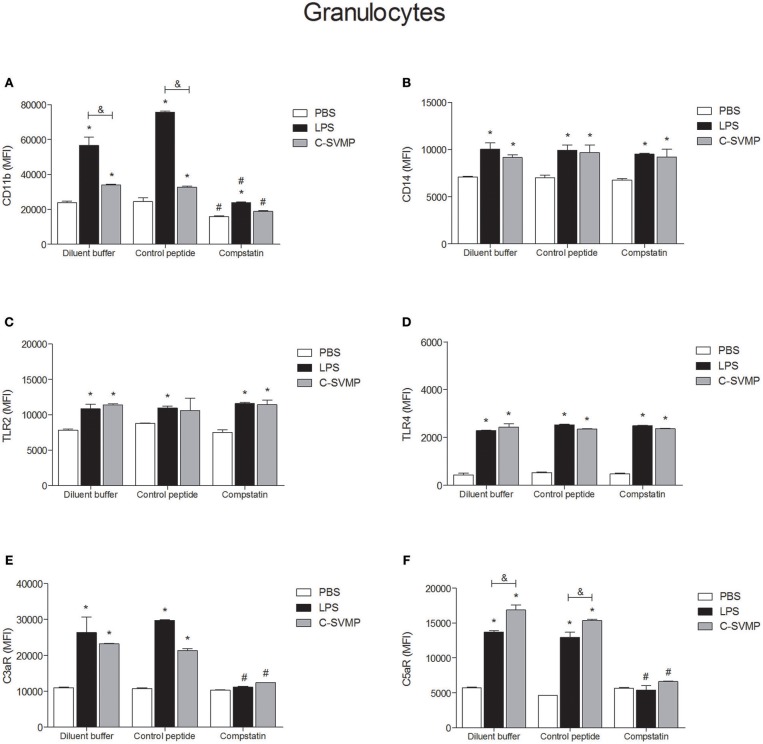
Partially complement-dependent activation of granulocytes by C-SVMP in the human whole blood model. Blood samples were collected in the presence of lepirudin and pre-incubated with diluent buffer, compstatin or control peptide for 30 min. Then, PBS, LPS or C-SVMP was added, and the samples were further incubated for 30 min. After erythrocyte lysis, leukocytes were fixed, stained with specific mouse anti-human monoclonal antibodies or isotype controls that were fluorochrome labeled and incubated for 30 min. Samples were analyzed by flow cytometry. Granulocytes were gated based on their forward- and side-scatter features, followed by selection of the CD66b^+^ population. The expression of CD11b **(A)**, CD14 **(B)**, TLR2 **(C)**, TLR4 **(D)**, C3aR **(E)**, and C5aR1 **(F)** was determined by the median fluorescence intensity (MFI) of stained cells after subtraction of the respective isotype control. Data represent the mean ± S. D. of four independent experiments. *p < 0.05 compared to the respective PBS treatment. &*p* < 0.05 between the LPS and C-SVMP treatments. #*p* < 0.05 between the compstatin and diluent buffer pretreatments.

Similar to what was observed in the monocyte population, blood granulocytes were also activated by C-SVMP, which enhanced the expression of CD11b, CD14, TLR2, TLR4, C3aR, and C5aR1 by these cells (Figure 3). The activation promoted by C-SVMP was comparable to that induced by LPS (Figure 3), except for CD11b, whose expression was higher in LPS-treated than in C-SVMP-treated blood (Figure 3A), and for C5aR1, whose expression was higher in C-SVMP-treated blood than in LPS-treated blood (Figure 3F). Pre-incubation with compstatin completely inhibited the upregulation of CD11b by C-SVMP in contrast to partial inhibition of this molecule in LPS-treated blood (Figure 3A). Complete inhibition of C3aR (Figure 3E) and C5aR1 (Figure 3F) upregulation was also observed in both treatments. The expression of CD14, TLR2 and TLR4 by granulocytes was not affected by pre-incubation with compstatin (Figures 3B–D).

### C-SVMP Induces the Partially Complement-Dependent Release of Pro-Inflammatory Cytokines and Chemokines in Human Whole Blood

**Figure 4:**
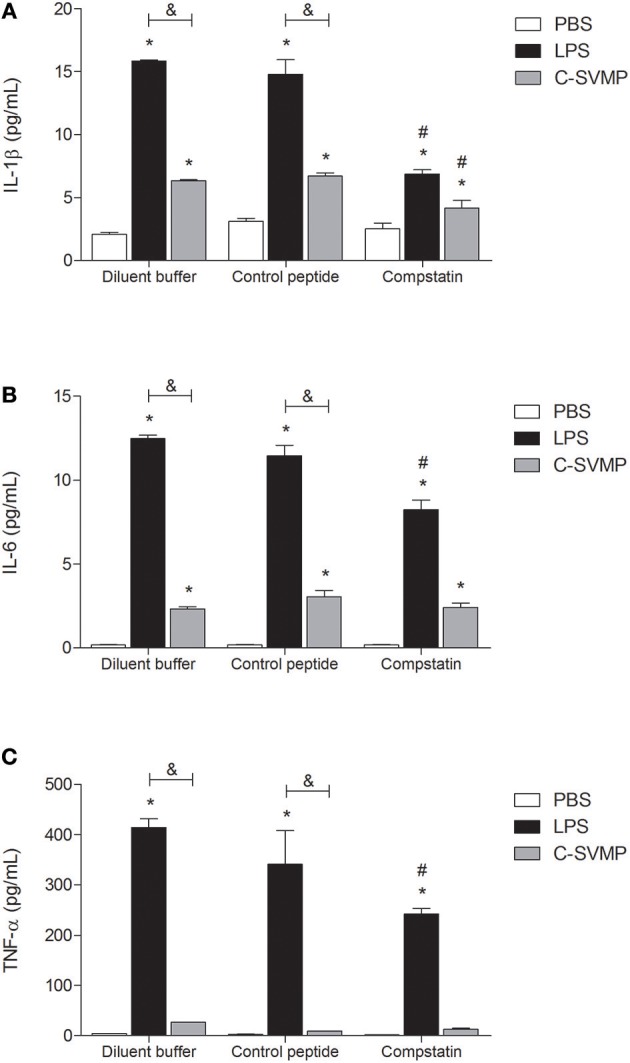
Partially complement-dependent release of pro-inflammatory cytokines in C-SVMP-treated human whole blood. Blood samples were collected in the presence of lepirudin and pre-incubated with diluent buffer, compstatin or control peptide for 30 min. Then, PBS, LPS, or C-SVMP was added, and the samples were further incubated for 30 min. Plasma levels of IL-1β **(A)**, IL-6 **(B)**, and TNF-α **(C)** were determined using the BD^TM^ Cytometric Bead Array (CBA) Human Inflammatory Cytokines Kit according to the manufacturer's instructions. Data represent the mean ± S. D. of four independent experiments. *p < 0.05 compared to the respective PBS treatment. &*p* < 0.05 between the LPS and C-SVMP treatments. #*p* < 0.05 between the compstatin and diluent buffer pretreatments.

Although at a lower degree than LPS, C-SVMP was able to induce detectable levels of IL-1β (Figure 4A) and IL-6 (Figure 4B) in human whole blood *in vitro*. The same effect was not observed for TNF-α release, which was induced only by LPS treatment (Figure 4C). Pre-incubation with compstatin partially affected the release of the three cytokines by LPS (Figure 4) but only affected the release of IL-1β by C-SVMP (Figure 4A).

**Figure 5:**
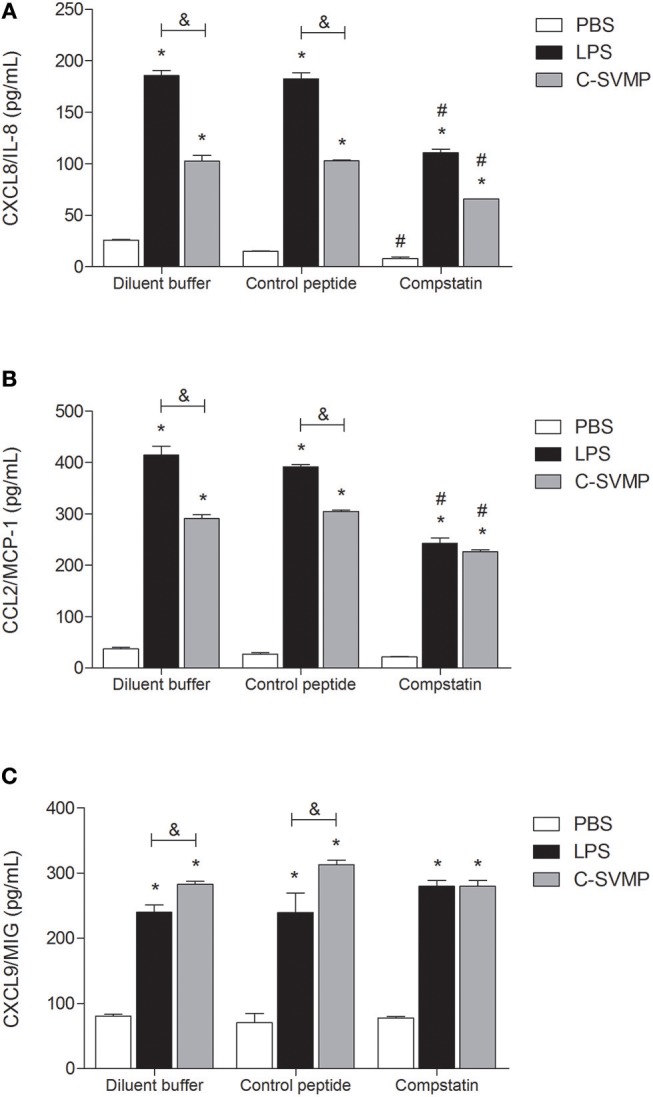
Partially complement-dependent release of pro-inflammatory chemokines in C-SVMP-treated human whole blood. Blood samples were collected in the presence of lepirudin and pre-incubated with diluent buffer, compstatin or control peptide for 30 min. Then, PBS, LPS, or C-SVMP was added, and the samples were further incubated for 30 min. Plasma levels of CXCL8/IL-8 **(A)**, CCL2/MCP-1 **(B)** and CXCL9/MIG **(C)** were determined using the BD^TM^ Cytometric Bead Array (CBA) Human Chemokine Kit according to the manufacturer's instructions. Data represent the mean ± S. D. of four independent experiments. *p < 0.05 compared to the respective PBS treatment. &*p* < 0.05 between the LPS and C-SVMP treatments. #*p* < 0.05 between the compstatin and diluent buffer pretreatments.

C-SVMP also induced an increase in the production of chemokines, such as CXCL8/IL-8 (Figure 5A), CCL2/MCP-1 (Figure 5B) and CXCL9/MIG (Figure 5C). Again, the C-SVMP and LPS effects were slightly different since C-SVMP treatment induced lower levels of CXCL8/IL-8 (Figure 5A) and CCL2/MCP-1 (Figure 5B) but higher levels of CXCL9/MIG (Figure 5C) compared to those induced by LPS treatment. Compstatin partially blocked the release of CXCL8/IL-8 (Figure 5A) and CCL2/MCP-1 (Figure 5B) by both stimuli and had no effect on the production of CXCL9/MIG (Figure 5C). IL-12p70, IL-10, IP-10, and RANTES were not detected in our assays (data not shown).

## Discussion

The complement system has been implicated in the pathogenesis of several inflammatory diseases through the generation of anaphylatoxins (C3a, C4a, and C5a), resulting from the cleavage of the C3, C4, and C5 components (21). In addition to other functions, anaphylatoxins induce (a) the release of vasoactive mediators by mast cells; (b) the chemoattraction of neutrophils and monocytes; (c) the stimulation of the oxidative burst; and (d) the release of pro-inflammatory cytokines by leukocytes (22). Based on the roles of anaphylatoxins in inflammation, complement inhibitors have been introduced as drug candidates for the treatment of complement-dependent inflammatory diseases (21).

The complement system is an important target of many toxins found in animal venoms (4). It is therefore reasonable to think that complement inhibitors could be used as complementary therapy in the treatment of accidents in which venom is able to activate the complement system. However, to our knowledge, there are few works in the literature suggesting this possibility, and only three really developed experiments using complement inhibitors to minimize the effects of animal venoms or toxins. In 2007, Mizuno et al. (23) showed that the complement inhibitor sCR1 (a recombinant soluble complement receptor type 1) was able to decrease the renal damage caused by a *Phyllodiscus semoni* sea anemone toxin. In 2013, the complement inhibitor eculizumab (humanized monoclonal anti-C5 antibody) was used in *in vitro* experiments to impair the complement-mediated hemolysis caused by *Loxosceles reclusa* venom (24). More recently, our group described the experimental use of a complement inhibitor, compstatin, to prevent complement activation by the main toxin from *Loxosceles* spider venom, the sphingomyelinase D (20). The present work is the first study using a complement inhibitor to control the effects of a toxin isolated from *Bothrops* snake venom.

Particularly in bothropic accidents, the inflammatory events caused by the venom involve, in addition to other mechanisms, the activation of the complement system (25). Thus, the main objective of the present work was to experimentally demonstrate that complement inhibitors could be useful as a complementary therapy in the treatment of bothropic accidents, especially in the control of the systemic inflammatory events caused by the venom.

The human whole blood model described by Mollnes et al. (14) is an appropriate human *ex vivo* model to study the role of the complement system in systemic pathologies, such as endotoxic shock (14, 16). This model was shown to be useful for the study of the systemic inflammatory events induced by animal venoms, as demonstrated by our group using *Loxosceles* spider venom (20). However, in the case of *Bothrops* venoms, there is a complicating factor, that is, their *in vitro* pro-coagulant effect due to the presence of thrombin-like enzymes, factor II activators and factor X activators, in addition to other pro-coagulant toxins (26).

Faced with the inability to work with the *Bothrops* whole venom in this model, we decided to study an isolated toxin with activity on human complement and without pro-coagulant activity. Previously, our group purified a P-I SVMP from *B. pirajai* venom and demonstrated its activation properties on human complement (6). The so-called C-SVMP did not promote blood coagulation under the conditions established in this work, allowing its use in the human whole blood model.

Although our group had already demonstrated the ability of C-SVMP to activate human complement *in vitro*, this effect had been observed with the use of normal human serum or purified complement components but not in the context of whole blood. Therefore, we first aimed to evaluate whether C-SVMP was able to significantly activate the complement system in the human whole blood model. For this purpose, we analyzed the levels of anaphylatoxins and the TCC generated in whole blood treated with C-SVMP, comparing with the levels in negative and positive control samples treated with PBS and LPS, respectively. C-SVMP was able to activate the complement system in the whole blood model, generating C3a/C3a desArg, C5a/C5a desArg and SC5b-9, thus confirming the previous results of our group (6). Interestingly, although both LPS and C-SVMP have induced similar levels of C5a/C5a desArg, C-SVMP induced lower levels of SC5b-9. A possible explanation for this phenomenon could be the cleavage of terminal pathway components by C-SVMP, thus impairing the formation of SC5b-9. Other snake venom metalloproteinases have already been implicated in the cleavage of terminal components. Atrase B, a class III metalloproteinase from *Naja atra*, was shown to cleave factor B, C6, C7 and C8, inhibiting the haemolytic activity of complement (27). A recombinant fibrinogenase from *Agkistrodon acutus*, named rFII, which contains the conserved catalytic triad of zinc-dependent metalloproteinases (28), was also able to degrade human C5, C6, and C9, decreasing the activity of complement (29).

Once we demonstrated the activation of the complement system by C-SVMP in whole blood, we showed that the complement inhibitor compstatin completely blocked the effects of C-SVMP on the generation of C3a/C3a desArg and SC5b-9 but only partially blocked the generation of C5a/C5a desArg (Figure 1). As compstatin was used at a concentration 3.17 times greater than the IC_50_ for the classical pathway and 16.67 times greater than the IC_50_ for the alternative pathway, we do not believe that this residual generation of C5a/C5a desArg was a consequence of insufficient compstatin but probably of direct cleavage of C5 by C-SVMP. In fact, C-SVMP is able to directly cleave the α chain of the human C5 molecule, generating C5a (6). Interestingly, the cleavage of C5 α chain by C-SVMP was shown to be more efficient than the cleavage of C3 and C4 α chains since incubation of 3 μg of human C3, C4, or C5 purified components with 0.32 μg of C-SVMP resulted in ~30–35% residual C3 and C4 α chains compared to ~20% residual C5 α chain (6). Therefore, considering that compstatin inhibits the cleavage of C3 by C3 convertase (17) and that the site of C3 cleavage for C-SVMP is the same as that for C3 convertase (6), it is reasonable to suggest that compstatin could also inhibit the direct cleavage of C3 by C-SVMP, while leaving C5 molecules free to be cleaved by C-SVMP. This hypothesis could explain the residual generation of C5a/C5a desArg even in the presence of compstatin.

Another mechanism that could explain the maintenance of C5a/C5a desArg detection in the presence of compstatin is the cleavage of C5 by proteases released by activated leukocytes. The description of such proteases is not recent. The cleavage of human C5 by neutrophil lysosomal granule lysate and the consequent generation of chemotactic fragments was first described in 1970 (30) and later confirmed (31). Non-canonical cleavage of C5 by leukocyte elastase does not occur at the same site as canonical cleavage by C5 convertase since elastase favors Val, Ser, Leu, or Ala amino acids at the P1 site, while C5 convertase cleaves at Arg74 (31). The region adjacent to the canonical site of cleavage is rich in amino acids susceptible to elastase cleavage, generating a functionally active C5a-like fragment (31), which probably cross-reacts with the C5a/C5a desArg antibodies used in our ELISA assay. Recent works have also described other non-canonical mechanisms for the intra- or extracellular cleavage of C5 by endogenous proteases, such as granzyme B, cathepsin D and elastase, with the generation of active fragments (32). Some of these proteases can be released upon leukocyte stimulation, as in the case with LPS- or C-SVMP-treatment in our model, thus justifying the persistence of C5a/C5a desArg detection even in the presence of compstatin.

Nevertheless, the residual non-canonical C5 cleavage did not reflect in the maintenance of SC5b-9 levels in our model, since the generation of SC5b-9 in response to C-SVMP or LPS treatment was decreased in the presence of compstatin. Some events may be concomitantly contributing to this phenomenon. First, as we have already mentioned above, cleavage of terminal pathway components could impair the formation of SC5b-9. Similarly to atrase B (27) and rFII (29), neutrophil elastase has also been shown to cleave the C6 component (31), which could impact the formation of TCC even in the LPS-treated samples. Another possible explanation could be that, in the presence of compstatin, only non-canonical C5 cleavage by leukocyte proteases or C-SVMP would occur. The C5b-like fragments generated through this non-canonical cleavage could be less active than the canonical C5b fragment, resulting in lower levels of TCC. In fact, (31) demonstrated that the non-canonical cleavage of C5 by human neutrophil elastase generates a C5b-like fragment less active and more unstable than C5b. The C5 cleavage by rFII also seems not to generate an active C5b fragment, since the human C5, C6 and C9 components were largely degradated by rFII, inhibiting the hemolytic activity (29). Finally, it is also important to note that, although the SC5b-9 levels have been decreased with the use of compstatin, they still were higher than the levels of C5a/C5a desArg in all compstatin-treated groups (934 ng/mL of SC5b-9 vs. 61 ng/mL of C5a/C5a desArg in PBS-treated samples; 1751 ng/mL of SC5b-9 vs. 98 ng/mL of C5a/C5a desArg in LPS-treated samples; 1078 ng/mL of SC5b-9 vs. 96 ng/mL of C5a/C5a desArg in C-SVMP). TCC levels are expected to be higher than C5a, due to differences in their half-life, which are about 60 minutes for TCC and 1 minute for C5a (33). Together, all these data suggest that, in our model, compstatin is able to inhibit the canonical complement pathways, but not the non-canonical ones. However, these non-canonical pathways seems to be less relevant, once they are able to generate lower levels of C5a-like fragments and less active C5b-like fragments.

In addition to allowing us to evaluate the activation of the complement system and its modulation by specific inhibitors, the human whole blood model also allowed us to verify the leukocyte activation pattern by assessing the expression of cell surface molecules and the release of pro-inflammatory mediators. C-SVMP was shown to exert an important pro-inflammatory effect in the whole blood model, upregulating the expression of CD11b, CD14, TLR2, TLR4, C3aR, and C5aR1 by monocytes and granulocytes and increasing the release of IL-1β, IL-6, CXCL8/IL-8, CCL2/MCP-1, and CXCL9/MIG. In *in vivo* experiments conducted by another group, C-SVMP (under the name of BpirMP) was able to increase the release of TNF-α, IL-6, and IL-10 and also showed oedematogenic effects and induced local leukocyte recruitment (11).

At least two mechanisms could be involved in the pro-inflammatory effects of C-SVMP in the whole blood model, which are the direct action or the indirect action on leukocytes, through the activation of the complement system or other endogenous mediators. Aiming to confirm these two possibilities, we evaluated the expression of cell surface molecules and the release of pro-inflammatory mediators in the presence of compstatin. In general, compstatin was able to inhibit the upregulation of CD11b, C3aR, and C5aR1 induced by LPS or C-SVMP in monocytes and granulocytes, indicating that the upregulation of these molecules were indirect effects of LPS and C-SVMP via the activation of the complement system. In contrast, the upregulation of CD14, TLR2, and TLR4 was not impaired by compstatin, demonstrating that these effects of C-SVMP and LPS were not complement-dependent. The release of cytokines and chemokines was only partially inhibited by compstatin.

Upregulation of CD11b by monocytes and granulocytes is an important hallmark of leukocyte activation in the human whole blood model (16). CD11b is the α-chain of CD11b/CD18 integrin, also known as CR3, which mediates leukocyte adhesion and migration during inflammatory processes and works as a complement iC3b receptor, playing a key role in the phagocytosis of iC3b-opsonized particles (34). The use of compstatin in the whole blood model aimed to demonstrate that CD11b upregulation mediated by C-SVMP is complement-dependent, as has already been demonstrated for LPS (16, 35). C3aR and C5aR1 were also shown to be complement-regulated by C-SVMP in this model. C3aR and C5aR1 are anaphylatoxin receptors expressed by some cell types, especially leukocytes, whose surface levels are upregulated in the presence of LPS (36). These receptors are essential for the response of leukocytes to complement activation since C3a and C5a act through their specific receptors to induce the inflammatory response (37). Therefore, our data suggest that this pathway of complement-dependent leukocyte activation is an important inflammatory pathway in C-SVMP-treated samples. Thus, blocking this pathway could be useful to impair the harmful inflammatory events in bothropic accidents.

In addition to other functions, signaling through C3aR or C5aR1 positively or negatively regulates signaling through TLRs, and vice versa, in a mechanism known as complement-TLR crosstalk (38). However, in our model, the crosstalk did not seem to be the predominant mechanism by which C-SVMP induced the upregulation of TLRs since their surface levels were not affected by compstatin. TLR2 and TLR4 are pattern recognition receptors (PRRs), which recognize pathogen-associated molecular patterns (PAMPs) such as lipoteichoic acid and LPS, respectively (38). Binding of lipoteichoic acid and LPS to TLR2 and TLR4, respectively, is not complement-mediated, but, through the crosstalk mechanism via CR3, for example, complement products can positively regulate the signaling of TLR2 and TLR4 (38). Signaling through these receptors could lead to their up-regulation in the cell membrane. Therefore, complement could be an important factor in the up-regulation of TLR2 and TLR4, even in LPS-treated samples. However, the use of compstatin indicated that, in this model, the up-regulation of TLR2 and TLR4 is not dependent on this crosstalk mechanism. CD14, which functions as a co-receptor for TLRs, especially in the recognition of LPS (39), was also unaffected by compstatin. All together, these results suggest that the complement-TLR crosstalk mechanism, which involves the complement system, TLRs and related molecules, such as CD14, is not the main mechanism by which C-SVMP induces the up-regulation of TLRs and CD14. These results implicate that other mechanisms, such as the direct binding of C-SVMP to cell receptors, or the production of endogenous ligands by the enzymatic action of C-SVMP, could occur. Direct binding of venom toxins to cell receptors has already been demonstrated for the venom of *Tityus serrulatus* scorpion and the production of endogenous ligands has also been proposed by the authors (40). In a recent review, Gutiérrez et al. (41) discussed the action of SVMPs on the extracellular matrix and the generation of biologically-active peptides, which could be associated with further tissue damage. The activity of SVMP on plasma proteins, such as fibrin and fibrinogen, are largely studied, what can also helps to corroborate the hypothesis of generation of endogenous ligands.

Signaling through innate cell surface receptors, such as C3aR, C5aR1 and TLRs, generally culminates in the activation of transcription factors, such as NF-κB, and increased production of pro-inflammatory cytokines and chemokines, including IL-1β, IL-6 and TNF-α (38). In our model, C-SVMP induced lower levels of IL-1β, IL-6, CXCL8/IL-8, and CCL2/MCP-1 than those induced by LPS, and it did not induce the release of TNF-α. These differences may be explained by the lower concentration of C-SVMP used in the assays compared to that of LPS. CXCL9/MIG was the only chemokine that showed higher levels in C-SVMP-treated blood compared to those in LPS-treated blood. CXCL9/MIG is a T cell chemoattractant usually produced by monocytes stimulated with IFN-γ (42). Compstatin was not able to impair the production of CXCL9/MIG induced by C-SVMP and only partially affected the production of the other mediators, indicating that the C-SVMP-induced release of cytokines and chemokines is probably only partially complement-dependent.

We thus hypothesize that C-SVMP could be directly recognized by PRRs, inducing several inflammatory effects in a complement-independent manner. Alternatively, soluble and/or cell surface endogenous molecules could undergo enzymatic action by C-SVMP, generating products with pro-inflammatory effects. In addition, it is important to remember that our results indicated the maintenance of non-canonical C5a generation even in the presence of compstatin, which could also contribute to the maintenance of CD14 and TLR expression as well as cytokine and chemokine release.

The set of our results does not allow us to exclude any of these hypotheses but allows us to state that at least part of the inflammatory effects of C-SVMP is complement-mediated and can be controlled by the use of complement inhibitors. The present work helps to open new perspectives for the use of complement inhibitors as complementary therapy in the treatment of accidents by venomous animals.

## Data Availability

All datasets generated for this study are included in the manuscript and/or the supplementary files.

## Ethics Statement

This study was approved by the Human Research Ethics Committee from the Biomedical Sciences Institute of the University of São Paulo (São Paulo, SP, Brazil) under the certificate number 1145/CEP.

## Author Contributions

LL performed all the experiments, analyzed the data and discussed the results. GP carried out the chromatographic runs for C-SVMP purification and discussed the results. CS-B standardized the gate strategies for flow cytometric analysis, discussed the results and wrote the manuscript. DT conceived the project, discussed the results and wrote the manuscript. All authors read, revised and approved the submitted manuscript.

### Conflict of Interest Statement

The authors declare that the research was conducted in the absence of any commercial or financial relationships that could be construed as a potential conflict of interest.
