# Corrigendum: Complement System Inhibition Modulates the Pro-Inflammatory Effects of a Snake Venom Metalloproteinase

**DOI:** 10.3389/fimmu.2019.01539

**Published:** 2019-07-03

**Authors:** Lygia Samartin Gonçalves Luchini, Giselle Pidde, Carla Cristina Squaiella-Baptistão, Denise V. Tambourgi

**Affiliations:** Immunochemistry Laboratory, Butantan Institute, São Paulo, Brazil

**Keywords:** *Bothrops pirajai*, SVMP, complement system, whole blood model, compstatin

In the original article, there was an error. The molar concentrations of LPS and C-SVMP, used in the assays, were incorrect.

A correction has been made to the **Materials and Methods** section, subsection **Human Whole Blood Model**, paragraph two:

“The human whole blood model described by Mollnes et al. (14) was used. Blood samples from healthy consenting donors were collected by venepuncture into tubes containing 50 μg/mL of lepirudin (Refludan®, Celgene, NJ, USA), the recombinant form of hirudin, a thrombin-inhibitor anticoagulant that does not interfere with the complement cascade. Immediately after collection, 720 μL of the blood samples were incubated with 280 μL of PBS (8.1 mM Na_2_HPO_4_, 1.5 mM KH_2_PO_4_, 137 mM NaCl, 2.7 mM KCl, pH 7.4), LPS (lipopolysaccharide from *Escherichia coli* strain O111:B4, Sigma-Aldrich, MI, USA; 5 μM final concentration) or C-SVMP (1 μM final concentration) for 30 minutes in a water bath at 37°C under agitation. Under these conditions, C-SVMP did not promote blood coagulation (data not shown), allowing for subsequent analyses of plasma and leukocytes, as described below”.

A correction has also been made to the **Materials and Methods** section, subsection **Blood Treatment With Compstatin or Control Peptide**, paragraph two:

“Herein, 720 μL of the blood samples were pre-incubated with 140 μL of diluent buffer (PBS), compstatin (Tocris Bioscience, Bristol, UK; 200 μM final concentration) or control peptide (Tocris Bioscience; 200 μM final concentration) for 30 min at 37°C. Then, 140 μL of PBS, LPS (5 μM final concentration) or C-SVMP (1 μM final concentration) were added and incubated for 30 min in a water bath at 37°C under agitation. Plasma and leukocytes were processed for analysis, as described below”.

The authors apologize for this error and state that this does not change the scientific conclusions of the article in any way. The original article has been updated.

